# Mechanical Quantification of Molecular Interactions Reveals Spatiotemporal Fluctuations on the Cell Surface

**DOI:** 10.1021/jacs.6c09095

**Published:** 2026-09-10

**Authors:** Sajan Shakya, Zhilei Zhang, Payton Ciolli, Arpit Sharma, Grinsun Sharma, Deepak Karna, Pravin Pokhrel, Jiahao Ji, Manabu Kurokawa, Yaorong Zheng, Hanbin Mao

**Affiliations:** † Department of Chemistry and Biochemistry, 4229 Kent State University, Kent, Ohio 44242, United States; ‡ School of Biomedical Sciences, 142731 Kent State University, Kent, Ohio 44242, United States; § Department of Biological Sciences, Kent State University, Kent, Ohio 44242, United States

## Abstract

The cell membrane hosts initial interactions between receptors and ligands for subsequent cellular functions. However, how the membrane spatiotemporally regulates binding mechanics in living cells remains unclear. Using magnetic tweezers, we developed a single-molecule platform to reveal that dissociation forces of integrin–RGD (arginylglycylaspartic acid), nucleolin–AS1411 (a nucleolin-binding DNA aptamer), and membrane–cholesterol complexes correlated with their respective binding affinities on HeLa cell surfaces. By quantifying the mechanical force that breaks molecular interactions on the live cell surface, we showed that lipid rafts have a stronger retainment of cholesterol than the rest of the membrane. Similarly, integrin–RGD binding is strengthened in lipid rafts, likely due to the preferential location of different integrin subtypes. Significantly, cell extensions show weaker integrin–RGD binding than the main bodies, consistent with the role of cell protrusions in migration, while increased integrin–RGD binding on cell main bodies indicates their critical role in cell anchoring. In addition, integrin–RGD binding is more strengthened during the S than the G1 phase of cell cycles, suggesting a higher priority for cells to focus on DNA synthesis in the S phase unperturbed while reducing cell migration in search of resources, an energy-costly process frequently adopted in the G1 phase. Our work provides a broadly applicable platform to interrogate nanomechanics of receptor–ligand interactions on the cell surface. These nanomechanical results indicate that the strength of molecular interactions on the cell surface fluctuates spatiotemporally to accommodate cell activities.

## Graphical abstract

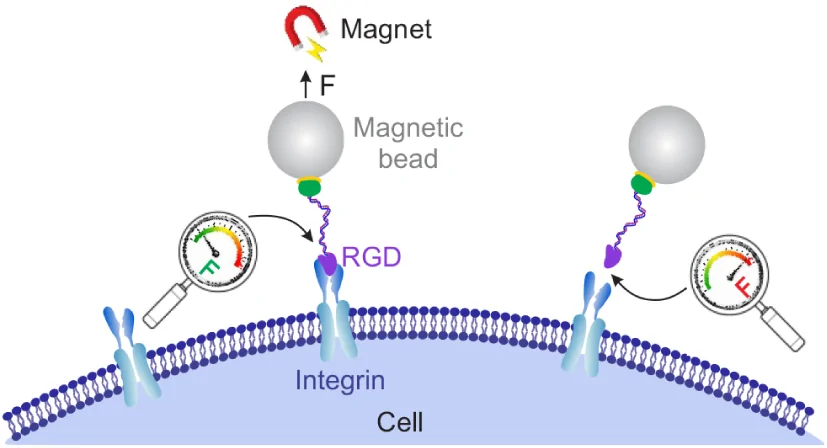

## Introduction

Molecular interactions on the cell surface are the first steps leading to numerous cellular processes, including adhesion, migration, and signaling.^1^ Despite their central importance, a fundamental physical parameter of these interactions remains largely unexplored in living systems: the mechanical strength of individual receptor–ligand bonds in their native cellular context. While biochemical and imaging approaches reveal where and when interactions occur, they do not report how strongly single-molecular complexes are mechanically stabilized on live cell membranes. Consequently, molecular binding strength is typically treated as a fixed molecular property, even though cells operate in mechanically heterogeneous and dynamically reorganizing membrane environments. These interactions are sensitive to the local cellular context, such as membrane heterogeneity, receptor clustering, and cytoskeletal coupling.^2^ Among diverse receptor families, integrins represent a unique class of transmembrane proteins, since their biological functions are tightly regulated by mechanical factors such as membrane pressures and extensile force experienced by cells.^3^ This makes them an ideal model for probing force-dependent receptor–ligand interactions,^4–6^ key phenomena in the mechanobiology and mechanochemistry fields.

The plasma membrane is a laterally heterogeneous structure, enriched with nanoscale lipid raft domains composed of cholesterol, sphingolipids, and proteins.^7^ These domains serve as organizing platforms that cluster receptors, regulate ligand accessibility, and modulate downstream signaling. Disruption of lipid rafts, for example, via cholesterol depletion with methyl-β-cyclodextrin (MβCD), can alter receptor distributions and binding dynamics, yet their precise force signatures remain unclear.^8^ In addition, whether membrane heterogeneity also regulates the mechanical stability of individual receptor–ligand bonds across different membrane regions remains unknown. The lack of tools capable of mapping molecular binding mechanics directly on living cell membranes has prevented quantitative evaluation of this fundamental question.

Single-molecule force spectroscopy techniques, such as atomic force microscopy, optical tweezers, and magnetic tweezers, have enabled direct quantification of the binding forces between individual receptor–ligand pairs.^9,10^ Among these, magnetic tweezers offer a unique combination of long-term measurement stability, high throughput, and minimized optical interference to probe interactions in situ and in real time on the cell surface. Previous magnetic tweezers studies have quantified force-dependent ligand–receptor dissociation in model systems and examined receptor-linked cell mechanics in living cells.^11^ However, these studies generally investigated functionalized surfaces or cellular mechanical responses following force application, rather than directly resolving rupture-force distributions for different molecular interactions on intact living-cell membranes. Consequently, despite the widespread use of magnetic tweezers in mechanobiology, their application as a platform for qunatitative single-molecule ligand–membrane force profiling on live-cell surfaces remains largely unexplored. While force-dependent dissociation of integrin–RGD (arginine-glycine-aspartic acid) pairs has been explored in reconstituted systems,^12^ their mechanical profiles on live-cell membranes, particularly in the context of spatial heterogeneity, membrane microdomain organizations, and temporal variations, remain poorly understood.

Here, we establish, to the best of our knowledge, the first magnetic-tweezers-based single-molecule force-mapping platform to directly quantify receptor–ligand mechanics on live cell membranes. Using integrin–RGD, nucleolin–AS1411 (a DNA aptamer), and membrane cholesterol interactions as model systems, we systematically examine how membrane microdomains, cellular location, and cell cycle stage regulate molecular binding mechanics. Whereas integrins exist as multiple subtypes that may be differentially partitioned into lipid rafts, nucleolin–AS1411 provides a biologically distinct raft-associated ligand–receptor system lacking known receptor subtypes. This comparison enables us to distinguish whether membrane microdomains directly modulate bond mechanics or instead act indirectly by redistributing the heterogeneous receptor populations. Our results reveal that lipid rafts selectively strengthen specific molecular interactions and that the binding strength varies across membrane regions and cell cycle states. By systematically probing protein-peptide, protein-nucleic acid, and lipid-membrane interactions, this work establishes a comprehensive framework for mapping single-molecule binding strength in native cellular environments. Collectively, our findings demonstrate that molecular bond mechanics are not fixed biochemical constants but dynamically regulated cellular variables that fluctuate across space and time to support distinct cellular functions. This provides broad implications for cell adhesion, signaling, and targeted therapeutic design.

## Results and Discussion

### Single-Molecule Mechanical Profiling of Molecular Interactions on the Cell Surface

We investigated the interaction between cell-surface receptors and ligands using a single-molecule magnetic tweezers instrument. Specifically, we probed the integrin receptor and RGD tripeptide interactions, which are critically involved in cell motions.^14^ To this end, we synthesized a 1558-bp dsDNA handle functionalized with an RGD molecule on one end and a biotin at the other (see SI Section S4 for detailed synthesis). The biotin label facilitates the interaction of the dsDNA handle with a streptavidin-coated superparamagnetic bead (2.32 μm Dynabeads M270, Sigma-Aldrich) whereas the RGD moiety in the construct binds to integrin on the HeLa cell surface (Figure 1A, inset).^15^

**Figure 1:**
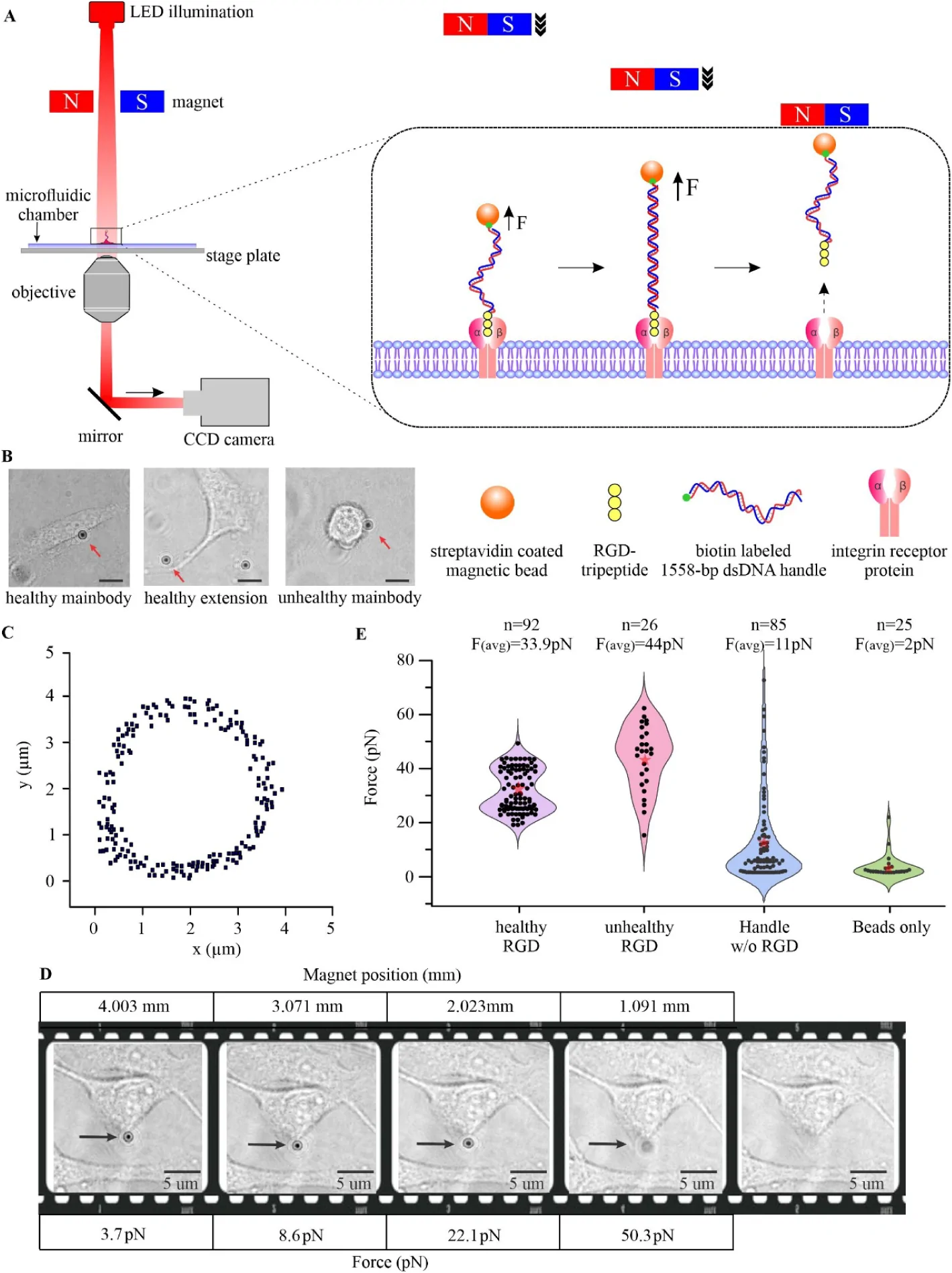
Magnetic-tweezers-based single-molecule force measurement of integrin–RGD interactions on HeLa cells. (A) Schematic of the magnetic-tweezers assay. A dsDNA handle (1558 bp) is functionalized with an RGD ligand and attached to a 2.32 μm streptavidin-coated superparamagnetic bead. The RGD motif binds to integrin receptors on the cell membrane. An external magnetic field lifts the magnetic bead, allowing measurement of the force required to disrupt the interaction between RGD and integrins on the cell surface. (B) Representative bright-field images of DNA-bound superparamagnetic beads attached to different cellular regions. Left: bead tethered to a healthy cell main body; middle: bead attached to a healthy cell extension; right: bead attached to an unhealthy main body. Arrows highlight beads of interest. Scale bars = 5 μm. (C) Quantification of bead rotation used to confirm the single DNA tether attached to a cell, showing a circular trajectory characteristic of single-molecule tethering.^13^ (D) Sequential bright-field images of a superparamagnetic bead (indicated by the black arrow) attached to the surface of a healthy HeLa cell in the microfluidic chamber inside the magnetic tweezers instrument. When a pair of magnets was brought closer to the chamber (vertical distance between the magnets and the chamber surface is marked at the top of each frame, see Figure 1A for setup), a tensile force was exerted on the magnetic bead (the magnitude of the force exerted at a specific distance is shown at the bottom of each frame). Diffraction rings of the magnetic bead changed as the bead was pulled away from the focal plane. (E) Violin plots of rupture forces to disrupt integrin–RGD interactions. Healthy cell extensions (purple, n = 92 cells) show a mean rupture force of 33.9 pN, whereas unhealthy cells (pink, n = 26) display a higher average force (44 pN). In contrast, dsDNA handles without the RGD ligand (blue, n = 85) and bare beads (green, n = 25) show nonspecific interactions with significantly lower forces (F_avg_ = 11 pN and 2 pN, respectively). The stars indicate average forces.

During the mechanical probing experiment, the beads conjugated with the DNA construct were flowed into a microfluidic chamber (see SI Figure S2) where HeLa cells were pre-grown for 5 h (see SI Section S7 for details). This allowed the dsDNA handle on the magnetic beads to bind with integrin receptors located on different cell surface regions such as the main body and extensions via integrin–RGD affinity interactions (Figure 1A and B). After 5 h, the majority of HeLa cells were alive, as confirmed by cell morphology visualization and/or live-and-death assay^16^ under a fluorescence microscope (see SI Section S2, Figure S1). Force was applied to the integrin–RGD binding complex by lowering down a pair of magnets placed over the microfluidic chamber (see close-up image in Figure 1A),^200,201^ which lifted the magnetic beads away from the cell surface while stretching the dsDNA handle. Single-molecule dsDNA tethers were confirmed by the rotary motion of tethered magnetic beads in the *xy*-plane, while each dsDNA tether was maintained at ∼4 pN (Figure 1C). In contrast, the magnetic beads tethered by multiple dsDNA strands showed irregular and asymmetric movements.^13,14^ Once single-tether attachment was confirmed, we then applied the tensile force to these single-dsDNA-tethered magnetic beads by lowering the magnets at a force-ramping rate of 0.7 pN/s (see SI Section S7 for details), a value observed within the loading-rate range of cell movements.^17^ When the applied pulling force exceeded the threshold to maintain the integrin–RGD interaction, the magnetic beads detached from the cell surface and diffused away from the focal plane (see Figure 1D). This detachment force was recorded as the rupture force.

After these integrin–RGD rupture forces were plotted as violin diagrams (Figure 1E), we found that the average rupture force in healthy cells (33.9 pN, n = 92) was smaller than that in unhealthy cells (44 pN, n = 26). Such an observation is likely due to compromised membrane integrity in unhealthy cells, which present sticky cell surfaces caused by excreted biomacromolecules such as DNA, RNA, and proteins, among others.^18^ For the following experiments, we focused only on healthy cells to avoid these implications. To confirm that the rupture observed in healthy cells was due to the disruption of integrin–RGD interaction, we performed control experiments with bare beads or those coated with a 1558-bp dsDNA handle without the RGD moiety (Figure 1E). We observed much lower average rupture forces (2 pN, n = 25; and 11 pN, n = 85, respectively) compared to the RGD-containing tethers, confirming that the higher forces (33.9 pN) were indeed attributable to the dissociation of the integrin–RGD complex in healthy cells. In the case of a handle without RGD control, the rupture events observed in high-force regions likely arise from a combination of nonspecific handle-cell interactions, membrane tether formation, glycocalyx-associated interactions, and other weak nonspecific adhesions at the cell surface.

### Effect of Lipid Raft on the Interactions between Ligands and the Cell Membrane

Given that the dynamics of receptor–ligand interactions are often regulated by lipid raft microdomains on the cell surface,^20^ we next examined whether lipid rafts can affect mechanical interactions in the integrin–RGD complex. We hypothesized that if integrin–RGD interactions preferentially occur in lipid rafts, then disrupting lipid rafts may weaken these interactions. To test this hypothesis, lipid rafts were compromised by treating HeLa cells with 10 μM methyl-β-cyclodextrin (MβCD), an agent known to disrupt lipid rafts by extracting cholesterol molecules critical for maintaining lipid rafts^21^ (Figure 2A). When we compared rupture forces of integrin–RGD interactions between cells with and without MβCD treatment (Figure 2B), we observed two force populations (42.7 and 26.5 pN) in cells without MβCD treatment (i.e., lipid raft intact cells). We surmised that the two rupture force populations in intact cells may represent two types of integrin–RGD interactions that occur either inside or outside lipid rafts. When we applied 10 μM MβCD to the cells, we found that the high-force population observed in untreated cells was no longer clearly resolved. In addition, the lower-force population shifted from 26.5 pN in untreated cells to 30.2 pN after MβCD treatment, and this difference was statistically significant (*p* = 2 × 10^–4^). In comparison, the difference between the 30.2 pN population and that of the high-force population (42.7 pN, see Figure S11 C) before MβCD treatment is even more significant, with *p* = 1.35 × 10^–18^. The shifted force indicates that MβCD treatment remodels the overall integrin–RGD force landscape. Among various effects of MβCD on integrin–RGD binding,^22^ the change in cell mechanics may play an important role. It is well-known that extraction of cholesterol by MβCD decreases membrane stiffness, which cushions against the force transmission from the DNA tether to the integrin–RGD binding complex, increasing the apparent rupture force to break this complex. Our force measurements on the integrin–RGD complex indeed revealed increased rupture force after incubation of HeLa cells in the presence of PFOA (perfluorooctanoic acid, see Figure S12), a compound that is known to soften cell membranes.^23^ The fact that lipid rafts present a higher rupture force (42.7 pN vs 26.5 pN) for the integrin–RGD interaction suggests that lipid rafts can strengthen these ligand–receptor interactions even with the same receptor type. Such a result is consistent with the finding that lipid rafts increase the binding affinity of ligands to receptors located within.^24^ Alternatively, different rupture force populations can be explained by the presence of different subtypes of integrins, which may bind to RGD with different affinities. Research has indeed found that different integrin subtypes prefer to locate inside or outside lipid rafts,^25^ which may lead to different strengths of RGD binding as observed here.

**Figure 2:**
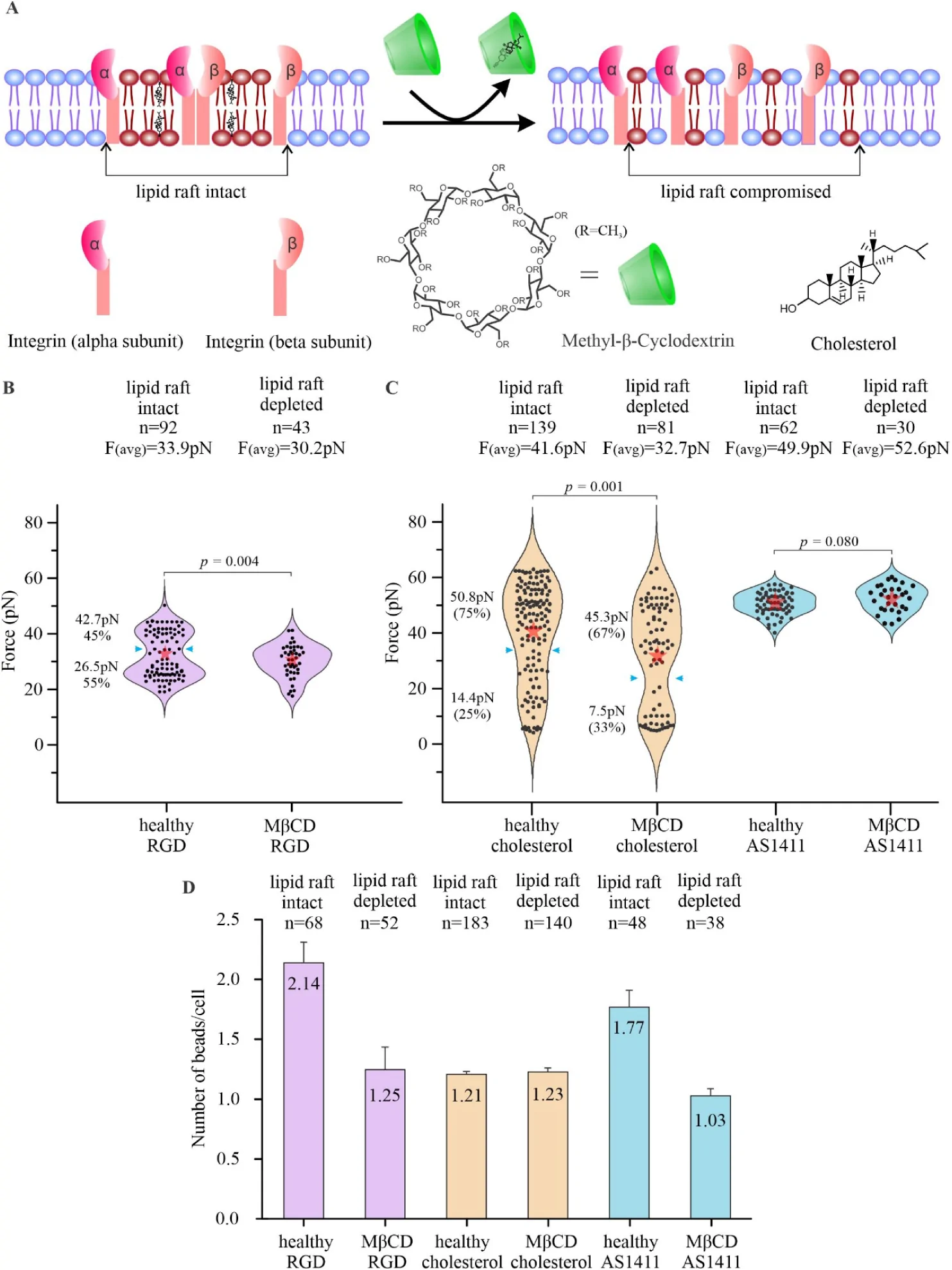
Effect of lipid rafts on the interaction between ligands and cell-surface receptors. (A) Schematic showing the role of lipid rafts in integrin clustering. In intact membranes (left), integrin α and β subunits are colocalized within cholesterol-rich lipid rafts, ready to bind RGD ligands. Treatment with MβCD (right) extracts membrane cholesterol and disrupts raft domains, leading to integrin dispersion and altered force profiles.^19^ (B) Violin plots of rupture forces between integrin–RGD interactions on healthy cells with intact lipid rafts (left, n = 92 cells, F_avg_ = 33.9 pN) and on cells with compromised lipid rafts after treatment with 10 μM methyl-β-cyclodextrin (MβCD) (right, n = 43, F_avg_ = 30.2 pN). Stars depict average values, whereas arrowheads represent the cutoff values separating different populations in (A) and (B). (C) Violin plots of rupture forces for membrane–cholesterol interactions on healthy and MβCD-treated cells (left two plots: n = 139, F_avg_ = 41.6 pN and n = 81, F_avg_ = 32.7 pN, respectively) and nucleolin–AS1411 complex on healthy and MβCD-treated cells (right two plots: n = 62, F_avg_ = 49.9 pN; and n = 30, F_avg_ = 52.6 pN, respectively). (D) Quantification of the average number of beads bound per cell for different ligands under lipid raft-intact and MβCD-treated (lipid raft-compromised) conditions. While cholesterol-functionalized beads showed minimal change in the average number of beads bound per cell, a significant reduction was observed for RGD- and AS1411-functionalized beads after lipid raft was compromised by MβCD. “n” represents the number of cells. Error bars depict the standard deviations.

To differentiate these two scenarios, we performed two control experiments. In the first control, we attached cholesterol to the end of the DNA strand using click chemistry (see SI Section S5 for detailed synthesis). Probing the cell membrane without MβCD treatment with this cholesterol-functionalized dsDNA construct (see SI Section S5, Figure S4) revealed two broad rupture-force populations that likely reflect the fact that cholesterol is a small hydrophobic molecule that can partition into many heterogeneous membrane microdomains and may also interact with some membrane proteins. The two rupture-force populations are 50.8 pN and 14.4 pN with a 75%:25% population ratio, respectively. This ratio significantly changed to 67%:33% (45.3 pN vs 7.5 pN) after cells were incubated with MβCD (Figure 2C, for the population change of 75% → 67%, *p* = 0.031). Since MβCD can disrupt lipid rafts, this result supports that the higher extraction-force population is likely associated with interactions occurring inside lipid rafts. This assignment was confirmed by the observation that the magnitude of the extraction force is significantly reduced for cholesterol in lipid raft-compromised cells with respect to those in lipid raft-intact cells (50.8 pN → 45.3 pN, *p* = 0.007). This is because lipid rafts show more lipid-ordered property compared to those outside lipid rafts,^26,27^ since it has been found that cholesterol prefers to lipid-ordered phase,^28^ the extraction force for cholesterol inside lipid rafts is expected to be larger. This experiment therefore demonstrated that our method can indeed probe lipid-raft domains by extraction-force measurements.

In the second control, we attached an AS1411 aptamer^29^ (see SI Section S6 for detailed synthesis) at the tip of the dsDNA handle. AS1411 is a G-quadruplex DNA aptamer^30,31^ that can bind to nucleolin, a membrane-associated protein preferentially located inside lipid rafts.^32^ Unlike integrin, however, there are no subtypes found for nucleolin.^33,34^ Therefore, the effect of lipid rafts on the distribution of integrin subtypes can be inferred from the force-mapping profiles of nucleolin. Using this nucleolin construct, we performed the same experiment described in Figure 1 to evaluate rupture forces between AS1411 and nucleolin on the HeLa cell surface with and without MβCD treatment (Figure 2C). We found that the rupture force of the nucleolin–AS1411 interaction did not significantly vary (49.9 → 52.6 pN, *p* = 0.080) after lipid raft was compromised by MβCD treatment. Such a result showed that the lipid raft environment is not sufficient to determine the strength of ligand-cell receptor interactions. Therefore, the different rupture-force populations observed to disrupt the integrin–RGD complex in Figure 2B may be due to different integrin subtypes located inside and outside lipid rafts, respectively.

To probe which integrin subtype is involved in RGD binding, we targeted αvβ5, one of the main integrin subtypes^35^ on the HeLa cell surface that have many different integrin expressions.^36^ We first performed immunostaining of HeLa cells using an anti-αvβ5 antibody. As shown in Figure S10 (SI Section S11), we indeed confirmed that this subtype was abundantly expressed on the HeLa cell membrane, which is likely responsible for the RGD binding observed here.

Additionally, we conducted the magnetic tweezers experiments with the anti-αvβ5 antibody. When we incubated the cultured cells in the magnetic tweezers chamber with the primary antibody against the αvβ5 integrin, we observed that the number of beads attached to each cell was reduced from 2.14 (n = 68) to 1.11 (n = 18) (*p* = 2 × 10^–5^) (Figure S11), likely due to occupancy of the antibody on that integrin subtype. In addition, we found that the higher force population observed in Figure 2B was significantly reduced (*p* = 0.006) (see Figure S11), suggesting that the higher-force population could be attributed to RGD binding to αvβ5, the main integrin subtype expressed on the HeLa cell surface.

Previously, single-molecule studies have demonstrated that integrin mechanics can vary substantially among receptor subtypes. For example, Jo et al. showed that α4β1 and the RGD-binding β1 integrins α5β1 and αVβ1 exhibit distinct force thresholds and cytoskeletal responses, indicating that integrin-mediated force transmission is strongly dependent on receptor subtype and ligand context.^37^ Our findings suggest that heterogeneous rupture-force distributions measured on living cell surfaces may reflect not only differences in membrane microenvironments but also variation in integrin subtype composition.

We found that average beads per cell was reduced for integrin–RGD and nucleolin–AS1411 interactions when HeLa cells were treated with MβCD (Figure 2D). This is consistent with the effect of MβCD to disrupt lipid rafts, in which both integrin and nucleolin are enriched. Disruption of lipid rafts disperses integrin and nucleolin, reducing the opportunity for the beads to attach to the cell surface through clustered receptors with more effective binding capabilities. On the other hand, this effect has little effect on membrane–cholesterol interactions as cholesterol is free to insert into membranes regardless of the presence of lipid rafts.

### Rupture Force Correlates with the Binding Strength of Ligand–Receptor Interactions

To correlate the rupture force with the strength of binding interaction between the ligand and the cell surface, we summarized all three interactions (integrin–RGD, nucleolin–AS1411, and membrane–cholesterol) in Figure 3. Since different lipid rafts have different compositions compared to the rest of the cell membrane, for clarity purpose, we focused on rupture-force events outside lipid rafts by applying MβCD. When we compared these rupture forces with dissociation constants estimated from the literature,^38–40^ we observed a correlation between average rupture forces and molecular binding strength outside lipid rafts (Figure 3). This correlation indicates that our force-probing method can qualitatively evaluate the strength of binding complexes in native biological environments that are difficult to measure using conventional techniques such as surface plasmon resonance and atomic force microscopy.^10,41^ Most of these approaches employ *in vitro* conditions that are oversimplified to avoid interference of macromolecules present in the biological milieu.

**Figure 3:**
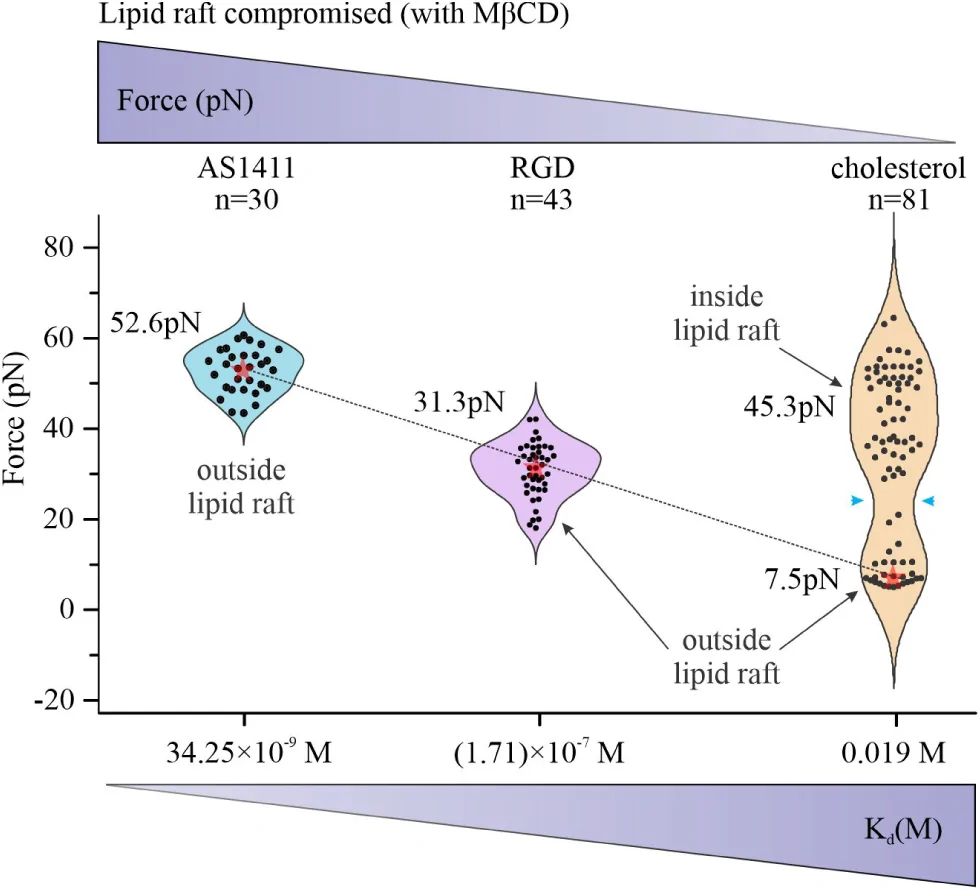
Molecular binding strength is correlated with rupture force. Violin plots showing rupture forces for nucleolin–AS1411, integrin–RGD, and membrane–cholesterol interactions in the presence of MβCD (i.e., with compromised lipid rafts). Higher forces are observed when binding interactions are stronger. Dissociation constants (K_d_) are taken from literature reports.^38–40^ The averaged K_d_ values have broad ranges since they were measured by different methods in different systems (see Table S6). The notation “n” represents the number of molecules measured. Stars depict the average rupture forces for the bindings outside the lipid rafts. For nucleolin–AS1411 interaction, the average rupture forces within and outside lipid rafts are similar. Blue arrowheads depict the cutoff points between high and low force populations. Dotted straight line is a guide to eyes for the populations outside lipid rafts.

In contrast, our approach enables *in situ* and real-time quantification of binding complexes ranging from a ligand bound to a macromolecular receptor, a macromolecule associated with another macromolecule, and even a small molecule interacting with the cell membrane itself. However, as rupture force is a kinetic variable that often depends on factors such as loading rate, receptor clustering, rebinding effects, membrane mechanics, and cytoskeletal coupling,^42,43^ caveat therefore should be given to directly correlate rupture forces of binding complexes with dissociation constants, thermodynamic terms that are largely not affected by the above factors. It should be noted that due to the kinetic aspect of the rupture force measurement, weak or short-lived interactions may dissociate before or during the force measurement cycle (∼2 min at 0.7 pN/s loading rate) and therefore may be underrepresented in the final rupture force distribution. This consideration is particularly important for integrin–ligand interactions because integrins can show force-dependent catch-bond and slip-bond behaviors.^44^ In catch-bond behavior, moderate tensile force can increase the lifetime of the integrin–ligand complex, whereas higher force can transition the interaction toward slip-bond-like dissociation. Future loading-rate-dependent or constant-force lifetime measurements will be useful for directly distinguishing catch-bond and slip-bond behaviors in force mapping on live cells by magnetic tweezers.

### Spatiotemporal Evaluation of Molecular Interactions on HeLa Cells

To demonstrate the capability of our mechanical approach to quantify the strength of ligand–membrane interactions on live cell surfaces, we evaluated whether the strength of the integrin–RGD interaction varies among different cell regions. For this purpose, we measured rupture forces from distinct cell regions: main bodies and cell extensions (Figure 4A and Table 1). For integrin–RGD interactions, the main body and cell extension exhibited no significantly different average rupture forces (34.8 pN, n = 56 for cell main bodies; and 33.2 pN, n = 41 for cell extensions, *p* = 0.161). However, the distribution of force subpopulations reveals a clear spatial variation.^34^ In the main bodies, the higher-force species, which corresponds to interactions occurring within lipid rafts (see Figure 2A), constituted 46% of the total population, whereas in the cell extensions, this high-force population significantly decreased to 39% (*p* = 0.020). This indicates that while the average binding force remains constant for the integrin–RGD complex, the population with stronger, lipid raft-associated interactions is higher on the main bodies. This is consistent with the role of lipid rafts in cell adhesions,^45^ in which the cell main body serves as the major adhesion point to the extracellular matrix.^46^ In contrast, since cell migration always starts at cell extensions,^47^ their adhering strength should not be as strong to facilitate reversible and repetitive detachment and attachment of integrin–RGD complexes for cells to move, which is essential for cells to search and take resources. This was indeed observed in Figure 4A where a decreased high-rupture-force population (39% at 41.1 pN) was observed on the cell extensions. Since TEM (Tetraspanin Enriched Microdomain) is relatively more resistant, whereas lipid raft is more susceptible to MβCD treatment,^48^ we surmise that the lower–rupture-force populations (26.8 and 26.6 pN, Figure 4A) remaining after MβCD treatment (see Figure 2A, right panel) are likely integrin subtypes located in the TEM, which is responsible for cell migrations. Therefore, the higher-rupture-force populations in the lipid raft can be ascribed to integrin subtypes, which allow cell adhesions and various signal transduction processes to occur.^49^ These two assignments are consistent with the fact that higher force is required to firmly adhere cells, whereas lower force is necessary for cell migrations to accommodate repetitive cell attachment and detachment (Figure 5).

**Figure 4:**
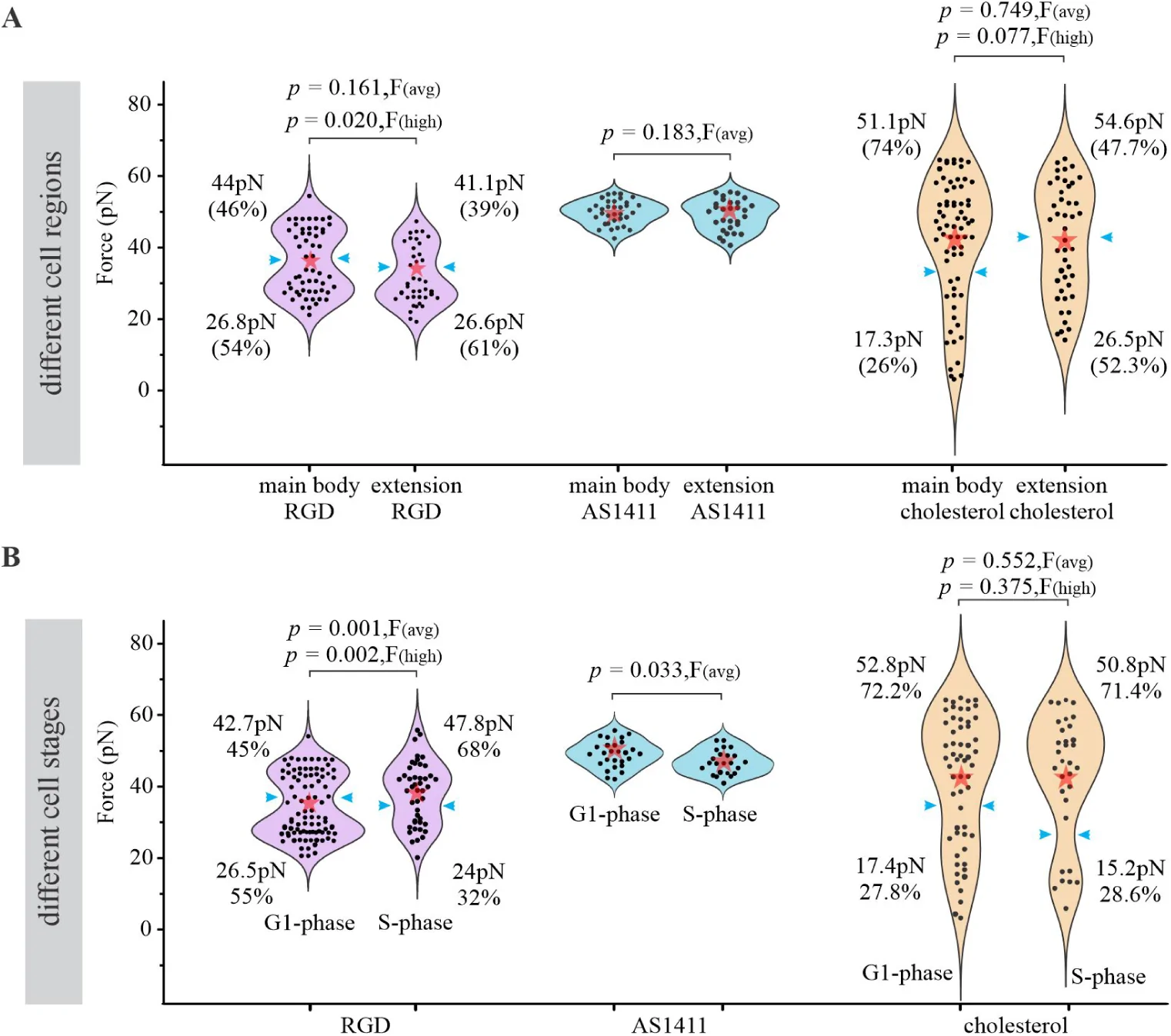
Spatial and temporal evaluation of ligand–membrane interactions on HeLa cells. (A) Violin plots showing rupture forces between ligand-functionalized DNA tethers and their interacting partners located on the cell main bodies or cell extensions. RGD, main body (n = 56 cells, F_avg_ = 34.8 pN), extension (n = 41, F_avg_ = 32.2 pN). AS1411, main body (n = 36, F_avg_ = 48.7 pN), extension (n = 30, F_avg_ = 50.2 pN). Cholesterol, main body (n = 69, F_avg_ = 42.2 pN), extension (n = 44, F_avg_ = 41.2 pN). Results indicate ligand-specific differences in force profiles across cell regions, with RGD showing enhanced binding in the main body (with more population inside lipid rafts (46%) with respect to that in cell extensions (39%), p = 0.020, see SI Section S9 for detailed calculation) whereas AS1411 and cholesterol showing similar bindings between the main bodies and the cell extensions (p = 0.183 and 0.749, respectively, for AS1411 and cholesterol). (B) Violin plots comparing rupture forces for the ligands on HeLa cells in G1 and S cell cycle phases. RGD, G1 phase (n = 92 cells, F_avg_ = 33.9 pN); S phase (n = 49, F_avg_ = 40 pN). AS1411, G1 phase (n = 26, F_avg_ = 50 pN); S phase (n = 22, F_avg_ = 47.6 pN). Cholesterol, G1 phase (n = 54, F_avg_ = 42.9 pN); S phase (n = 35, F_avg_ = 40.6 pN). Stars depict the average forces for different conditions. Blue arrowheads represent the cutoff points between high and low force populations. For populations that do not have an obvious cutoff point, we used Gaussian fitting to determine (see SI Section S9).

**Table 1:** Spatiotemporal Fluctuations of Molecular Interactions on the Cell Membrane

	Cell motion	Cell cycle regulator	Membrane integrity	
	Integrin–RGD	Nucleolin–AS1411	Membrane–Cholesterol	Notes
Spatial difference (main body *vs* extension)	Spatial remodeling of integrin–RGD mechanical states. (*p* = 0.020)	No strong spatial difference detected. (*p* = 0.183)	Spatially stable under current measurement conditions. (*p* = 0.077)	Facilitates resource taking
Temporal difference (G1 *vs* S phase)	Cell-cycle-associated remodeling of integrin–RGD mechanics. (*p* = 0.002)	Modest cell-cycle-associated change. (*p* = 0.033)	No significant cell-cycle-associated difference detected. (*p* = 0.375)	Facilitates energy expenditure

**Figure 5:**
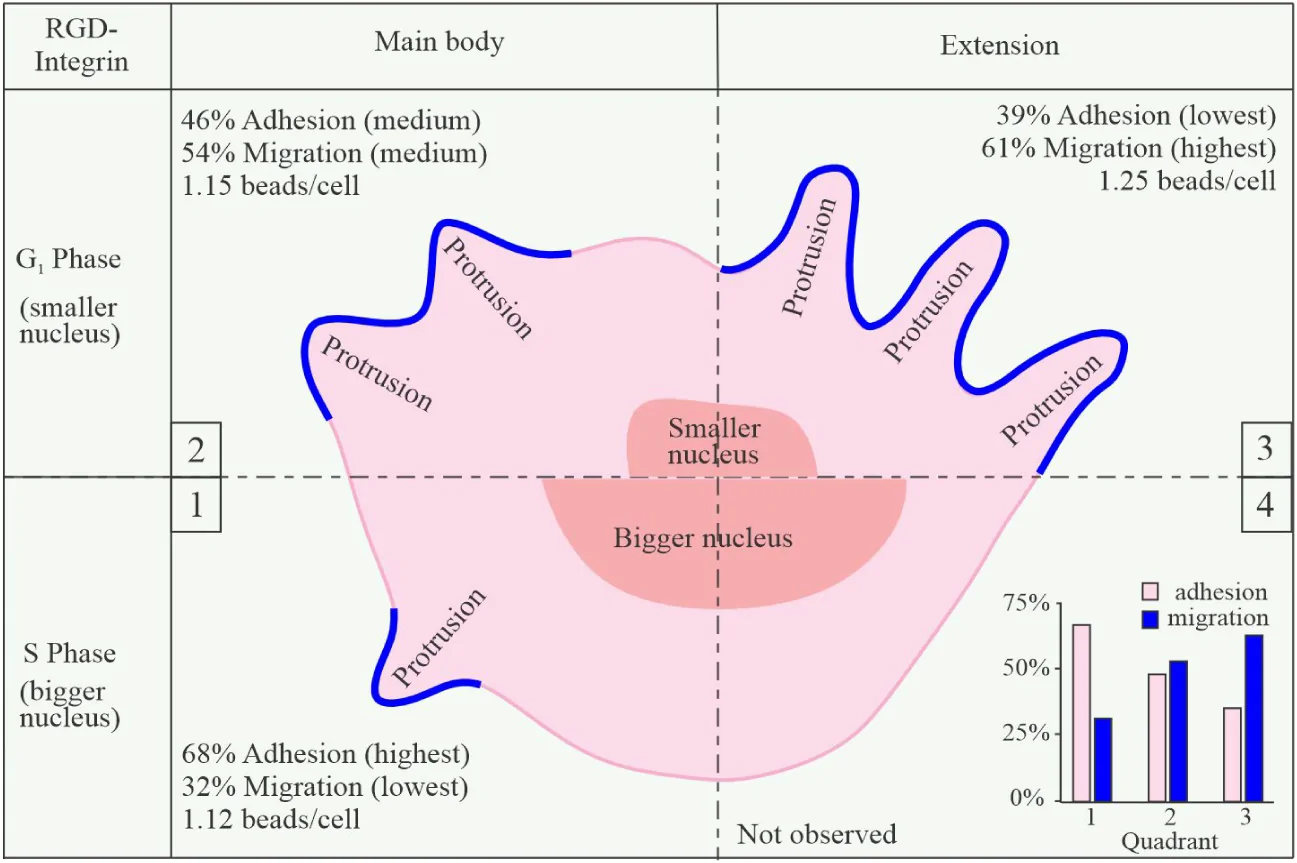
Schematic of integrin–RGD binding measured by magnetic tweezers on HeLa cells during G_1_ phase (smaller nucleus) and S phase (larger nucleus). In G_1_ phase, the main body (quadrant 2, 1.15 beads/cell) exhibits 46% integrin population for adhesion and 54% for migration (see SI Section S10 for calculations), whereas cell extensions (quadrant 3, 1.25 beads/cell) show reduced adhesion (39%) but enhanced migration (61%). In S phase, the main body (quadrant 1, 1.12 beads/cell) displays the highest adhesion (68%) and lowest migration (32%), while binding events in cell extensions (quadrant 4) were not observed. The inset summarizes the distribution of adhesion (pink box) and migration (blue box) across different cell regions and phases.

Previous studies have found that there are more integrins located at the edge of cell extensions.^50^ Our results supported this finding, as the integrin density was higher in cell extensions (1.25 beads per cell) than in main bodies (1.15 beads per cell). It is possible that these integrins in cell extensions are located in the TEM for cell movements unlike the main body integrins, which preferentially participate in high-affinity, lipid raft-associated adhesive interactions (Figure 4A).

On the other hand, both nucleolin–AS1411 and membrane–cholesterol demonstrated similar binding strengths for the subpopulations of the main bodies and cell extensions (see Figure 4A). For AS1411, the average rupture forces for main bodies and extensions are 48.7 pN and 50.2 pN (*p* = 0.183) respectivly. Likewise, for cholesterol, the average rupture forces for main bodies and cell extensions are 42.2 pN and 41.2 pN (*p* = 0.749), respectivly As both nucleolin and cholesterol are enriched in lipid rafts,^32^ these findings suggest that some compositions of the cell membrane, such as nucleolin and membrane lipids, do not vary in different cell regions, allowing them to maintain membrane integrity so that cells can sustain normal activities during migrations (Table 1).

Next, we used this force quantification method to measure the strength of the integrin–RGD interaction during different cell phases. HeLa cells exhibit four different cell phases: G1, S, G2, and M phases.^51^ We chose G1 and S phases as they can be easily distinguished by cell morphology^52^ and cell cycle analysis using a flow cytometer (for details, see SI Section S8), while their relatively longer durations allowed convenient force measurement with respect to G2 and M phases.^53^ As shown in Figure 4B and Table 1, we found that integrin–RGD binding in S phase has a significantly higher average rupture force (47.8 pN) compared to G1 phase (42.7 pN) with *p* = 0.002. In addition, significant difference in the percentage of the higher-force population between S phase (68%) and G1 (45%) (*p* = 0.002) (see SI Section S9 for detailed calculations). Since integrins are transmembrane receptors involved in cell-matrix attachment, these results suggest that a greater fraction of integrins is engaged in lipid-raft-associated, high-affinity states, which could reflect stronger but less dynamic adhesion sites in the S phase. Such a finding is consistent with the fact that the G1 phase is when cells search for and accumulate resources before DNA replication in the S phase.^54^ Therefore, the integrin–RGD complex should not be too strong to facilitate the movement of cells to search for nutrients. Once cells enter the S phase, it is more beneficial for cells to stay fixed^55^ (i.e., with a stronger affinity for the integrin–RGD complex), thereby preserving the energy to focus on DNA replications.^56^ The fact that both we and others^57^ observe very few cell extensions during S phase was consistent with this scenario (i.e., migration has a lower priority). From the perspective of TEMs (Tetraspanin Enriched Microdomains) and lipid rafts as described above, the G1 phase contains more TEM integrins responsible for cell migrations, consistent with its role for resource searching during the G1 phase; whereas the S phase has enriched lipid raft integrins, suggesting that the integrins responsible for cell adhesion and signal transductions, such as checkpoint signaling,^58^ are more important than those for cell migration during DNA synthesis in the S phase.

On the other hand, nucleolin–AS1411 interactions show a significantly higher force in the G1 phase (50 pN, see Figure 4B) than in the S phase (47.6 pN; *p* = 0.033). Nucleolin is a phosphoprotein whose expression, localization, and phosphorylation vary throughout the cell cycle.^59^ During the S phase, it has been found that nucleolin has decreased population on the cell surface after translocating to the nucleus for DNA replication.^60^ Such a reduction is therefore expected to shift the equilibrium to dissociate the nucleolin–AS1411 complex, reducing its rupture force.

In contrast, there is no significant difference at the 95% confidence level for the extraction-force populations of the membrane–cholesterol complexes between the G1 and S phases (*p* = 0.375 for the higher-force populations of 72.2% and 71.4%, respectively, Figure 4A). The relatively constant level of membrane–cholesterol interactions between the G1 and S phases suggests that a stable membrane framework is needed to maintain essential cellular functions.

## Conclusion

In summary, we developed and applied magnetic-tweezer-based single-molecule force profiling to directly quantify ligand–membrane interactions on live cell surfaces and to correlate their mechanical strengths with distinct cellular functions. By systematically probing integrin–RGD, nucleolin–AS1411, and membrane–cholesterol complexes, we showed that lipid-raft heterogeneity selectively modulates interaction strength in a manner that supports cell motion and membrane integrity. Integrin–RGD interactions function as a cell motion indicator, exhibiting strong spatial and temporal heterogeneity: reduced high-force populations in cell extensions facilitate reversible cell adhesions (i.e., cell migrations), which is important for resource acquisition during the G1 phase, while enhanced lipid raft-associated binding in the main body anchors cells, which is beneficial for cells to focus on DNA replication during the S phase (see Table 1). In contrast, nucleolin–AS1411 interactions, which participate in transmembrane transport and nucleic acid synthesis, remain spatially uniform but are temporally regulated through cell cycles. Finally, membrane–cholesterol interactions remain largely invariant across both space and time, underscoring their role in preserving membrane integrity. Collectively, these new findings not only demonstrate that cells dynamically tune specific ligand–membrane mechanics to accommodate changing functional demands but also establish that single-molecule force mapping is a broadly applicable framework to dissect receptor–ligand nanomechanics in native cellular environments.

## Materials and Methods

### Chemicals and Reagents

All chemicals and reagents with at least 99.9% purity, if not specifically mentioned, were purchased from Thermo Fisher Scientific, Waltham, MA. Enzymes were purchased from New England Biolabs (NEB), Ipswich, MA. DNA oligos (mentioned on Table S1) were obtained from Integrated DNA Technologies (IDT), Coralville, IA, and purified by PAGE (for more details, see Section S1, Supporting Information).

### Cells and Cell Culture

HeLa cell line was obtained from ATCC (American Type Culture Collection). HeLa cells (Human Cervical Cancer Cells) were cultured in DMEM (Dulbecco’s Modified Eagle’s Medium, Corning Incorporated, Corning, NY) supplemented with 5% FBS (Fetal Bovine Serum, Premium, Thermo Fisher Scientific, Waltham, MA) and 1% Penicillin-Streptomycin Solution (Corning Incorporated, Corning, NY). Cells were grown in a Thermo Scientific HERAcell Vios 160i CO_2_ incubator (Thermo Fisher Scientific, Waltham, MA) at 37 °C with 5% CO_2_ supply. Cells were passaged at a confluence of about 90%.

### Microfluidic Chamber Preparation

Magnetic tweezers experiments were conducted in a custom microfluidic chamber constructed by sandwiching a single-channel Parafilm spacer between two glass coverslips (see SI Section S3 for details). The bottom coverslip was coated with 0.003% poly-l-lysine (PLL) to facilitate surface attachment, while the top coverslip contained two access holes serving as the inlet and outlet ports for fluid exchange. This configuration allowed for controlled introduction and replacement of buffers during the experiments.

### Synthesis of Functionalized DNA Construct

Three functionalized DNA constructs, which include RGD construct, cholesterol construct, and AS1411 construct, were synthesized and purified in our laboratory (see SI Sections S4–S6 for details) and used for magnetic tweezers experiments. Each construct consisted of a 1558 bp double-stranded DNA (dsDNA) handle modified with a specific functional moiety at one end to enable interaction with cellular receptors or the cell membrane, while the opposite end contained a biotin label for attachment to streptavidin-coated magnetic beads. The RGD construct was used to probe integrin-mediated receptor–ligand interactions, the cholesterol construct enabled membrane anchoring through lipid insertion, and the AS1411 construct targeted nucleolin receptors on the cell surface.

### Single-Molecule Magnetic Tweezers Experiment

Prior to magnetic-tweezers measurements, HeLa cells were seeded in the microfluidic chamber and incubated to allow firm adhesion of the cells to the glass surface (see SI Section S7 for details). To minimize nonspecific interactions, the cells were first treated with salmon sperm DNA as a blocking agent to avoid nonspecific interactions. The cells were then incubated with the functionalized DNA constructs (see SI Sections S4–S6) and streptavidin-coated magnetic beads, enabling specific receptor–ligand attachment and formation of bead-cell tethers. Following incubation, the chamber was mounted on the magnetic tweezer setup, where desired magnetic forces were applied to the tethered beads while bead motion was monitored by optical microscopy. LabVIEW software was used for raw data acquisition, while PIMicroMove software controlled the piezo stage and magnet positioning to apply the programmed force-loading protocol.

## Supplementary Material



## Notes

Materials and reagents, HeLa cell culture and sample preparation, magnetic bead functionalization, magnetic tweezers force measurements, quantification of rupture-force, Gaussian population analysis and statistical methods, integrin–RGD interactions, cholesterol–membrane interactions, nucleolin–AS1411 interactions, lipid rafts and membrane microdomains, spatial heterogeneity of cell-surface interactions, cell-cycle-dependent temporal fluctuations, lipid-raft disruption by methyl-β-cyclodextrin, αvβ5 integrin characterization, control experiments, additional force distributions and statistical analyses, and references (DOCX)
