# Supplementary material for: Mechanical Quantification of Molecular Interactions Reveals Spatiotemporal Fluctuations on the Cell Surface
Source: J Am Chem Soc. 2026 Sep 10;148(44):47762–72. doi: 10.1021/jacs.6c09095 (PMC13761265; doi:10.1021/jacs.6c09095)
Supplement: Supplementary file 1 [file ja6c09095_si_001.docx]

**Supporting information**

**for**

**Mechanical quantification of molecular interactions reveals spatiotemporal fluctuations on cell surface**

Sajan Shakya^1^, Zhilei Zhang^1^, Payton Ciolli^2^, Arpit Sharma^1^, Grinsun Sharma^2^, Deepak Karna^1^, Pravin Pokhrel^1^, Jiahao Ji^1^, Manabu Kurokawa^2,3^, Yaorong Zheng^1,2^, Hanbin Mao^1,2,^ *

^1^ Department of Chemistry and Biochemistry, Kent State University, Kent, OH 44242, USA

^2^ School of Biomedical Sciences, Kent State University, Kent, OH 44242, USA

^3^ Department of Biological Sciences, Kent State University, Kent, OH 44242, USA

* Corresponding author: Hanbin Mao (hmao@kent.edu)

**Table of Contents**

[S1. List of oligos used in this work	3](#_Toc236083456)

[S2. Multimodal imaging of HeLa Cells	3](#_Toc236083457)

[S3. Microfluidic chamber	4](#_Toc236083458)

[S4. Synthesis of functionalized RGD-DNA construct	6](#_Toc236083459)

[S5. Synthesis of functionalized cholesterol-DNA constructs	8](#_Toc236083460)

[S6. Synthesis of functionalized AS1411-DNA constructs	9](#_Toc236083461)

[S7. Magnetic-tweezers experiment on HeLa cell surface	11](#_Toc236083462)

[S8. Identification of HeLa cell cycle stages based on morphology and flowcytometry assay	13](#_Toc236083463)

[S9. Chi square (χ^2^) value determination and calculation of *p*-value	15](#_Toc236083464)

[S10. Spatiotemporal compartmentalization of mechanical adhesion and migration across the cell cycle	20](#_Toc236083465)

[S11. Immunostaining assay for αvβ5 integrin	22](#_Toc236083466)

[S12. Influence of αvβ5 antibody blocking and cholesterol depletion on integrin-RGD mechanical interactions	24](#_Toc236083467)

[S14. Literature-derived K_d_ values used for qualitative comparison with magnetic tweezers rupture forces.	25](#_Toc236083468)

# **S1. List of oligos used in this work**

***Table S1.*** *List of oligos used in this work.*

| **Oligo ID (# of nts)** | **Sequence (5**′**-3**′**)** |
| --- | --- |
| BSaI HF oligo (30) | CAGGGACGCGCTGGGCTACGTCTTGCTGGC |
| Comp-BSaI HF oligo (31) | CGGTGCCAGCAAGACGTAGCCCAGCGCGTCC |
| AS1411-MT1 (33) | CGGTTTTGGTGGTGGTGGTTGTGGTGGTGGTGG |
| AS1411-MT2 (37) | GTCAGTGACCACCACCACCACAACCACCACCACCAAA |
| AS1411-MT3 (37) | TTTGGTGGTGGTGGTTGTGGTGGTGGTGGTCACTGAC |
| DBCO-oligo (25) | /DBCO-N/GGCTACACTAGAAGGACAGTATTTG |
| Comp-DBCO-oligo (29) | CGGTCAAATACTGTCCTTCTAGTGTAGCC |

# **S2. Multimodal imaging of HeLa Cells**

***Figure S1.*** *HeLa cells visualized under three imaging modes. Left: magnetic-tweezers view showing cell surfaces attached with superparamagnetic beads. Center: bright-field image highlighting overall HeLa cell morphology and bead distribution. Right: fluorescence image from a live/dead assay (see text below) confirming cell viability during the experiment. Scale bars = 10 μm.*

Figure S1 shows HeLa cells imaged using three complementary microscopy techniques used during the magnetic-tweezers experiment. In the bright field image (left), the dark circular dots represent superparamagnetic beads (2.32 μm, Dynabeads M270, Sigma Aldrich) attached either to the chamber surface nonspecifically or to the cell surface through receptor-ligand interactions. These beads allow magnetic force to be applied on the cell surface in the magnetic-tweezers instrument. The bright-field image (center) displays the overall morphology of the HeLa cells, confirming bead attachment locations on cell surfaces. The fluorescence image (right) shows a live/dead cell assay ^1^ (Invitrogen LIVE/DEAD Cell Imaging Kit 488/570, Thermo Fisher Scientific, catalog no. R37601) in which live HeLa cells emit green fluorescence, validating that the cells remain viable under experimental conditions (see SI section S7).

# **S3. Microfluidic chamber**

***Figure S2.*** *Schematic of a single-channel microfluidic chamber preparation.*

A single-channel microfluidic chamber was assembled using two cover glasses (length × width; 60 mm × 24 mm, VWR International, LLC, Radnor, PA) separated by a Parafilm M laboratory film (thickness, ~0.15 mm, Uline, Pleasant Prairie, WI). Two holes serving as inlet and outlet were drilled into the top cover glass using a laser cutter (Versa Laser VLS200, Scottsdale, AZ) to allow fluid exchange. A single straight channel (length × width; 52 mm ×3 mm, at center) was cut into a parafilm film, which served as the spacer and defined the dimension of the flow path. Both plain and holed cover glasses were first cleaned with a glass cleaner (Glass Plus, Ace Hardware, Oakwood Village, OH) and rinsed with UltraPure DNase/RNase-free distilled water (Invitrogen, Waltham, MA). They were then sonicated (Fisher Scientific FS30 Ultrasonic cleaner, 130 W) sequentially in DI water and 95 % ethanol (v/v, reagent/ACS grade; Thermo Scientific 190 proof 95% Reagent Ethanol) for 10 minutes each. After sonication, the chambers were rinsed with DI water and treated with piranha solution (3:1 ratio of 98% sulfuric acid, ACS reagent grade and hydrogen peroxide, 30 wt % in H₂O, Sigma-Aldrich, St. Louis, MO) in a glass container at 80 °C for 90 minutes. Following this, the cover glasses were cooled in air, rinsed again with DI water, and sonicated for 30 minutes in DI water, followed by sequential sonication for 30 minutes each in 50%, 70%, and 95% ethanol. The cover glasses were then air-dried under UV light (254 nm, power 12 watts) for 4-5 hours. Finally, the plain cover glasses were coated with 0.003% poly-*L*-lysine (P*L*L) solution (Sigma‑Aldrich Inc. St. Louis, MO) and allowed to air dry.

After both cover glasses were thoroughly cleaned and dried, two layers of Parafilm were overlapped and carefully placed on top of the holed cover glass. The plain cover glass coated with 0.003% w/v poly-*L*-lysine (P*L*L) was then gently placed over the parafilm-glass assembly. All steps were performed inside a laminar flow hood (1300 Series A2, Thermo Scientific, Marietta, OH, USA) to maintain sterility. The assembled setup was subsequently heated on a hot plate (Isotemp, Fisher Scientific, Pittsburgh, PA, USA) at 95 °C for 10-15 seconds, allowing the parafilm to melt and form a sealed barrier around the microchannel, which was approximately 52 mm in length, 3 mm in width, and ~0.3 mm in height. Finally, each chamber was wiped with 70% ethanol before use. The drilled holes in the cover glass served as inlet and outlet ports for introducing solutions to the microchannel, as illustrated in Figure S2.

# **S4. Synthesis of functionalized RGD-DNA construct**

A detailed flowchart for the synthesis of functionalized RGD (arginylglycylaspartic acid)-DNA construct is shown in Figure S3. Briefly, a 30-nts BsaI HF oligo (see sequence in Table S1) was first enzymatically modified to introduce a 5′-terminal thiol (-SH) group. This was achieved using T4 Polynucleotide Kinase (New England Biolabs, USA) in the presence of ATP-γ-S (Enzo Life Sciences, USA) at 37 °C for 1.5 hours, which enables transfer of a thiophosphate to the 5′ hydroxyl group, thereby generating a reactive 5′-thiol functionality. This 5′-SH-BsaI HF oligo was then reacted with maleimide-DBCO (Dibenzocyclooctyne) (Sigma-Aldrich, St. Louis, MO) at 37 °C for 2 hours to functionalize a DBCO moiety, which then underwent a Strain-Promoted Azide-Alkyne Cycloaddition (SPAAC) reaction with an azide moiety ^2^ in the RGD-azide peptide (Sigma-Aldrich, St. Louis, MO). The SPAAC reaction occurred at 37 °C for 24 hours resulting in an oligo bearing an RGD motif at its 5' end. The RGD-containing oligo was then hybridized with a 31-nt comp-BsaI HF oligo (see sequence in Table S1) by heating the mixture at 95 °C for 5 minutes, followed by a gradual cooling from 95 °C to 20 °C at a rate of -0.5 °C/minute. This hybridized product was ligated to a biotin-labeled 1558-bp dsDNA handle (see next paragraph for its preparation) by T4 DNA ligase at 16 °C for 18 hours, yielding the final construct with biotin at one end for anchoring on the surface of streptavidin-coated magnetic beads via streptavidin-biotin affinity interaction and RGD at the other end for binding with integrins on cell surface as shown in Figure 1A. Such a construct enables the application of mechanical force via magnetic tweezers to probe integrin-RGD interactions on cell membranes.

***Figure S3.*** *Synthesis of a single-molecule RGD-functionalized DNA construct for the study of integrin-RGD interaction in magnetic-tweezers instrument.*

The 1558-bp dsDNA handle was generated by PCR amplification of the pBR322 plasmid using a 5′-biotinylated primer to enable surface attachment (forward primer sequence: 5′ AAA ATC TAG AGG CTA CAC TAG AAG GAC AGT ATT TG 3′, reverse primer sequence: 5′ biotin-TEG-GCA TTA GGA AGC AGC CCA GTA GTA GG 3′). The resulting PCR product was purified using Qiagen PCR purification kit (Sigma-Aldrich, USA). The purified fragment was then digested with the BSai HF V2 restriction enzyme to create a defined sticky end that allowed specific and efficient ligation with the RGD-containing dsDNA prepared above. This step facilitated the construction of a stable and functional DNA assembly suitable for downstream single-molecule experiments.

# **S5. Synthesis of functionalized cholesterol-DNA constructs**

***Figure S4.*** *Synthesis of functionalized DNA construct.*

A detailed flowchart for the synthesis of functionalized cholesterol-DNA construct is shown in Figure S4. Briefly, A functionalized 5′-DBCO-oligo (an intermediate prepared in Figure S4) was conjugated to cholesteryl-TEG-azide (Vector Laboratories, Inc., Newark, CA) via SPAAC reaction at room temperature (23 °C) for 18 hours. The product was purified using a 3k DA molecular weight cut-off filter (Amicon, Sigma-Aldrich, St. Louis, MO, USA) to remove unreacted reagents. The purified click product (cholesteryl-DNA conjugate) was then annealed with a 5′-phosphorylated comp-DBCO-oligo (Table S1) to generate a dsDNA construct with a ligation-compatible sticky end. This duplex DNA was subsequently ligated to a biotinylated 1558 bp dsDNA handle (1:10 molar ratio) using T4 DNA ligase at 16 °C for 18 hours, and the ligation product was purified by agarose gel electrophoresis to isolate the desired cholesterol-DNA-handle conjugate.

# **S6. Synthesis of functionalized AS1411-DNA constructs**

A detailed flowchart for the synthesis of functionalized AS1411-DNA construct is shown in Figure S5. First, an oligo (AS1411-MT1, see sequence in Table S1) was enzymatically phosphorylated at its 5′ end using T4-PNK at 37° C for 3 hours to enable ligation. It was then annealed with a partially complementary strand (AS1411-MT2, see sequence in Table S1), creating a short duplex with a single stranded overhang. This duplex was ligated to a biotinylated 1558 bp dsDNA handle (see section S4 for its preparation) at a 10:1 molar ratio using T4 DNA ligase at 16 °C for 18 hours, forming a covalent link but leaving a nick in one strand. To remove the strand complementary to the AS1411 aptamer, a third oligo (AS1411-MT3, see sequence in Table S1) was annealed to hybridize with the strand complementary to AS1411-MT2 by heating at 95 °C, followed by slow cooling at the rate of -0.5 °C /min for 2.5 hours to enable rapid hybridization. Finally, the desired product was purified through Agarose gel purification to remove any unligated DNA or excess unreacted oligos, yielding a chimeric DNA construct consisting of a 1558 bp dsDNA handle, whose biotin end allowed its attachment to the superparamagnetic bead coated with streptavidin, and the AS1411 aptamer, which could bind to the nucleolin expressed on the HeLa cell membrane, for mechanical characterization using magnetic tweezers instrument.

***Figure S5.*** *Synthesis of functionalized AS1411-DNA construct for the study of nucleolin- AS1411 interaction.*

# **S7. Magnetic-tweezers experiment on HeLa cell surface**

***Figure S6.*** *Detailed process for magnetic tweezers experiments to probe molecular interactions on HeLa cell surface.*

For magnetic tweezers experiment on HeLa cells, around 10,000 HeLa cells were seeded into a clean and sterile chamber and incubated in HERAcell VIOS 160i CO₂ incubator (Thermo Scientific, Waltham, MA) for 5 hours to ensure proper cell attachment and growth. The cells were then treated with 3% Salmon sperm DNA (Rockland Immunochemicals, Inc., Limerick, PA) in complete DMEM media for 20 minutes to remove potential non-specific interactions that arise due to cell debris. This was followed by a brief incubation of 7 minutes with functionalized DNA constructs prepared above to tether magnetic beads to cell surface (3 µl magnetic bead with 0.3 mg/ml concentration, 3 µl DNA construct of 0.015 pM, and 94 µl of complete DMEM media). After incubation, the chamber was washed slowly with complete DMEM media to remove unattached beads, leaving only those specifically bound to the DNA constructs. Finally, the prepared cells were placed in the magnetic-tweezers instrument, where controlled magnetic forces were applied to the beads and bead displacements were monitored using microscopy.

To begin the pulling experiments using magnetic force, the chamber was carefully removed from the Petri dish and gently wiped with a Kimtech Science Precision Wipes (Kimberly-Clark Professional, Roswell, GA) to remove any residual culture media on the chamber. It was then mounted and securely fixed onto the bracket. The objective lens was adjusted to bring the superparamagnetic beads into focus, and the stage was moved to identify a region within the chamber where multiple beads were attached to the cell surface. To determine a single-molecule tether, a pair of cubic magnets, with a 0.5 mm gap between them, (5 mm NdFeB magnets coated with Nickel, Supermagnete, Germany) was rotated at a speed of one rotation per second. A symmetrical circular trajectory of the magnetic bead (Figure 1C) indicated a single DNA tether between the magnetic bead and the chamber surface ^3^. After confirming the single-molecule tether, a home-built LabVIEW program (National Instrument, TX) was used for raw data collection and the piezo stage (model P-545.xR8S) was controlled using PIMicroMove software (Physik Instrument (PI) GmbH & Co. KG, Karlsruhe, Germany) in such a way that magnet was lowered at a rate of 0.7 pN/sec (the loading profile was validated by converting magnet positions to forces using the calibrated force equation shown below within a specific time window). A red LED (λ = 660 nm; 13 mW, M660F1, Thorlabs, Newton, NJ) was used for illumination to track beads. Simultaneously, recording software (OBS Studio, Wizards of OBS LLC) captured the video frame when the magnetic bead was detached (see Figure 1D), which revealed a specific distance between the bead and the chamber surface (*d*). This detachment distance was later converted into the rupture force using the calibration equation provided below ^4^,

$$F=\left( -121840 \right)\times e^{-0.771\times d}+(121970)\times e^{-0.7711\times d}$$

where *F* is rupture force of the event (force required to detach a bead from cell surface), *d* is the distance between the bottom surface of the magnet and the top chamber surface at which the rupture event occurs.

# **S8. Identification of HeLa cell cycle stages based on morphology and flowcytometry assay**

HeLa cells at different stages of the cell cycle were distinguished based on their characteristic cellular morphology ^5^ observed under magnetic tweezer microscopy, as illustrated in Figure S7 A and B. Cells identified as G1 phase typically exhibited a relatively small and compact cell body, reflecting the early stage of cell growth following mitosis. In contrast, cells in the S phase appeared larger and more spread with increased cytoplasmic area, consistent with active DNA replication and increased biosynthetic activity ^6^. These morphological characteristics allowed us to visually differentiate G1 and S phase cells during the magnetic-tweezers experiments.

To further validate cell cycle staging, we performed flow cytometry analysis at different incubation times. For this, confluent HeLa cells were harvested in 1 mL PBS (Phosphate-Buffered Saline) (pH = 7.4) (Thermo Scientific, Waltham, MA) at two different incubation times (6 hrs and 8 hrs) and were centrifuged at 1800 revolution per minute (rpm) in a centrifuge (Marathon 8k; Fisher Scientific, Waltham, MA) for 3 min. Then supernatant was discarded and the resulting cell pellets were fixed by adding 1 mL of chilled 70% ethanol dropwise while gently vertexing to ensure uniform fixation and minimizing cell clumping. The fixed cells were then stored at -20 °C for 24 hrs for further treatment. After fixation, the cells were centrifuged at 4 °C for 5 min at 13,000 rpm in a centrifuge (Universal 32R Centrifuge; Hettich Zentrifugen, Tuttlingen, Germany), as ethanol-fixed cells require a higher centrifugal force for efficient pelleting. The supernatant was removed, and the cells were washed twice with 1 mL PBS followed by centrifugation under the same conditions. After removing the PBS, 4 µL of Ribonuclease A (RNase A, 2 mg/mL) (Roche Diagnostics GmbH; Mannheim, Germany) was added, and the samples were incubated at room temperature for 30 min to eliminate RNA. Subsequently, 5 µL of propidium iodide (PI, 10 mg/mL) (>94% HPLC, Sigma-Aldrich, St. Louis, MO) was added, and the samples were incubated for an additional 15 min to allow DNA staining. Flow cytometry analysis was then performed directly in the RNase A and PI-containing solution, and 10,000 events were acquired for each sample. For determination of cell cycle stages based on PI staining, the fluorescence signal was plotted with PE-A (Phycoerythrin-Area, a fluorescent dye) on the x-axis and counts on the y-axis, as shown in Figure S7 C and D. The results indicated that at 6 hours, the majority of cells were in the G1 phase; whereas after 8 hours, a significant fraction of cells progressed to the S phase (see Figure S7 C and D). Based on these findings, magnetic tweezers experiments were conducted at corresponding time points to populate G1 and S phase cells, respectively.

***Figure S7.*** *HeLa cells at different cell cycle stages. Cells in* *G1 phase* ***(A)*** *display a more compact cell bodies, whereas cells in S phase* ***(B)*** *appear larger and more spread, consistent with active DNA replications* ^6^*. These morphological features were used as visual references to assign cell cycle stages during magnetic tweezers experiments.* *Flow cytometry of HeLa cells after 6 hrs* ***(C)*** *and 8 hrs* ***(D)*** *(see the paragraph for details). After 6 hrs, most cells remain in G1 (41.63%), with smaller populations in S (27.87%). After 8 hrs, the G1 fraction decreases (18.17%) while the S-phase fraction increases (45.53%), indicating progression of cells from G1 into S phase. Scale bars: 10 µm. The cell area is highlighted by black dotted outline.*

# **S9. Chi square (**χ^2^**) value determination and calculation of *p*-value**

To determine the force threshold separating the high-force and low-force populations, the rupture force distributions were analyzed by Gaussian fitting. For each dataset, we compared 1-3 Gaussian-peak fittings. The best fitting was determined by reduced Chi square ($\chi_{\mathrm{red}}^{2}$) values^7^. After best fitting is set, the force thresholds are determined by the cross points of the two adjacent Gaussian population curves (represented by a black arrow, see Figure S8).

***Figure S8.*** *Deconvolution of rupture force populations observed in integrin-RGD (from Figure 2B) interaction during magnetic tweezers experiments with various curve fittings. (A)For healthy integrin-RGD, the two curve fitting shows the least reduced chi square value (5.342) as compared to one (19.725) and three (12.186) curve fitting, whereas (B) For MβCD treated the one curve fitting shows the least reduced chi square value (2.043) as compared to one curve fitting (3.745) (See Table S2). (C) Showing two curve fitting for membrane-cholesterol interaction in mainbody and extension region. Black arrow indicates the force threshold to separate the force populations.*

For the comparison between various fittings the reduced chi-square ($\chi_{red}^{2})$ value was calculated as,

$\chi_{red}^{2}$ = $\frac{\chi2}{N-p}$

where, $"\chi^{2}"$is the raw chi-square value, “*N*” is the number of fitted data points, and “*p*” is the number of adjustable parameters. For healthy integrin-RGD interaction, using 16 data points (bins from the histogram) and 4, 7, and 10 fitting parameters for the one, two, and three component models, respectively, the calculated reduced chi-square values were 19.725, 5.342, and 12.186 (See Table S2). Because a lower reduced chi-square indicates better agreement between the fitted model and the experimental data, the two-component model provided the best fit for healthy integrin RGD among the evaluated models. Similarly, for MβCD treated integrin-RGD interaction, using 18 data points (bins from the histogram) and 4 and 7 fitting parameters for the one and two component models, respectively, the calculated reduced chi-square values were 2.043 and 3.745. (See Table S2).

**Table S2:** Reduced Chi square ${(\chi}_{\mathrm{red}}^{2}$) value for healthy and MβCD treated integrin-RGD interaction under various peak fitting”

| Interaction | Fitting model | χ^2^ | Parameters | Degree of freedom | ${(\chi}_{\mathrm{red}}^{2}$ |
| --- | --- | --- | --- | --- | --- |
| Integrin-RGD (Healthy) | One curve | 236.700 | 4 | 12 | 19.725 |
|  | Two curve | 48.078 | 7 | 9 | 5.342 |
|  | Three curve | 73.117 | 10 | 6 | 12.186 |
| Integrin-RGD (MβCD) | One curve | 28.600 | 4 | 14 | 2.043 |
|  | Two curve | 41.191 | 7 | 11 | 3.745 |

For each subpopulation, the mean force and standard deviation (SD) were calculated independently. These means for three data subsets were treated as independent replicates (n = 3 per condition). Statistical comparison between experimental groups was performed using a two-tailed unpaired t-test with Welch’s correction (Welch’s t-test) ^8^, which does not assume equal variance between groups. The tests were applied to the overall populations as well as high-force subpopulations to evaluate whether a statistically significant difference existed between different conditions (see Table S3-S5).

Table S3. *p*-value calculation for Figure 2

|  | Integrin-RGD | | Membrane-cholesterol | | Nucleolin-AS1411 | |
| --- | --- | --- | --- | --- | --- | --- |
|  | w/o MβCD | w MβCD | w/o MβCD | w MβCD | w/o MβCD | w MβCD |
| Average Force (pN) | 33.9 | 30.2 | 41.6 | 32.7 | 49.9 | 52.6 |
| Standard deviation (SD) | 8.8 | 5.8 | 18.2 | 19.7 | 4.0 | 7.5 |
| *p*-value | 0.004 | | 0.001 | | 0.080 | |

Table S4. *p*-value calculation for Figure 4A

|  | | Integrin- RGD | | Membrane- cholesterol | | Nucleolin-AS1411 | |
| --- | --- | --- | --- | --- | --- | --- | --- |
|  |  | Main body | Extension | Main body | Extension | Main body | Extension |
| For the overall force population | Average force (pN) | 34.8 | 33.2 | 42.2 | 41.2 | 48.7 | 50.2 |
|  | Standard deviation (SD) | 9.4 | 7.9 | 17.2 | 15.9 | 4.0 | 4.5 |
|  | *p-*value | 0.160 | | 0.749 | | 0.183 | |
| For the high force sub-population | % high force population (%) | 46 | 39 | 74 | 47.7 | - | - |
|  | Average  force (pN) | 44 | 41.1 | 51.1 | 52.3 | - | - |
|  | Standard deviation (SD) | 3.9 | 3.5 | 8.3 | 6.5 | - | - |
|  | *p*-value | 0.020 | | 0.0778 | | - | |

Table S5. *p*-value calculation for Figure 4B

|  | | Integrin- RGD | | Membrane- cholesterol | | Nucleolin-AS1411 | |  |
| --- | --- | --- | --- | --- | --- | --- | --- | --- |
|  |  | G1 phase | S phase | G1 phase | S phase | G1 phase | S phase | |
| For the overall force population | Average force (pN) | 33.9 | 40 | 42.9 | 40.6 | 50.0 | 47.6 | |
|  | Standard deviation (SD) | 8.8 | 13.2 | 18.2 | 18.2 | 4.0 | 3.6 | |
|  | *p*-value | 0.001 | | 0.552 | | 0.033 | |  |
| For the high force sub-population | % high force population (%) | 45 | 68 | 72.2 | 71.4 | - | - | |
|  | Average high force (pN) | 42.7 | 47.8 | 52.8 | 50.8 | - | - | |
|  | Standard deviation (SD) | 4.1 | 8.0 | 8.6 | 9.2 | - | - | |
|  | *p-*value | 0.002 | | 0.375 | | - | |  |

For the integrin-RGD interaction, *p* = 0.002 for the high force populations (42.7 pN and 47.8 pN respectively for the G1 and S phases) and *p* = 0.031 for the low force populations (26.5 pN and 24 pN respectively for G1 and S phases). For the membrane-cholesterol interaction, *p* = 0.375 for the high force (52.8 pN and 50.8 pN respectively for the G1 and S phases) and *p* = 0.486 for the low force populations (17.4 pN and 15.2 pN respectively for G1 and S phases). In addition, there is no significant difference for the percentage force population between the G1 and S phases (*p* = 0.08 for the higher force populations and (*p* = 0.25) for the lower force populations).

# **S10. Spatiotemporal compartmentalization of mechanical adhesion and migration across the cell cycle**

To quantify spatiotemporally resolved mechanical behaviors, adhesion and migration, across the G1 and S cell stages, individual events were separated into two functional regions: the main body and the cell extensions. For each region, the average bead-cell interaction events were determined to evaluate regional differences in mechanical interactions. The events were further categorized into higher rupture force events (for adhesion) ^9^ and lower rupture force events (for migration) ^10^. The relative percentages of adhesion and migration were calculated by normalizing the number of the events in each category to the total number of interactions within the corresponding cell region or cell-cycle stage, as follows,

$$\%=\frac{\text{Number of events in }\text{a particular }\text{category}}{\text{Total events in }\text{a }\text{region }\text{or}\text{ stage}}\times100$$

For example, there were 97 rupture force events in the G1 stage. Among those, 56 events occurred in main body and 41 in cell extensions. Next, these rupture force events were classified into higher and lower rupture force regimes. For the main body, 30 are higher force rupture events and 26 lower rupture force events. Therefore, the percentage of higher force events, which correspond to cell adhesions was calculated as,

$$Adhesion \% =\frac{\text{Number of }\text{higher rupture force }\text{events }\text{in the main body}\text{ }}{\text{Total events in }\text{the main body}}\times100$$

$$\%=\frac{\text{30}}{\text{56}}\times100$$

$$\%=\text{54\%}$$

Similarly, the percentage of lower force events in main body, which correspond to cell migration, was calculated as,

$$Migration \% =\frac{\text{Number of }\text{lower rupture force }\text{events }\text{in the main body}\text{ }}{\text{Total events in }\text{the main body}}\times100$$

$$\%=\frac{\text{26}}{\text{56}}\times100$$

$$\%=\text{46\%}$$

Using these formula, the percentages of the adhesion (39%) and migration (61%) in cell extensions were calculated respectively. Similarly in the S phase, all the population belongs to G1 phase and respective percentage for adhesion (68%) and migration (32%) were calculated. (See Figure S9 for detailed calculation).

***Figure S9.*** *Percentage distribution of* *adhesion and migration events across various regions in different cell phases*

# **S11. Immunostaining assay for αvβ5 integrin**

For immunostaining of αvβ5 integrin on HeLa cells, around 10,000 HeLa cells were seeded onto a clean and sterile glass coverslips coated with 0.003% w/v P*L*L (See section S3 for details) and incubated in HERAcell VIOS 160i CO₂ incubator (Thermo Scientific, Waltham, MA) overnight to ensure proper cell attachment and growth. Then, the cells were washed twice with 1x PBS to remove detached cells and were fixed by incubating at 37 °C for 30 minutes with 4% Paraformaldehyde (PFA) in PBS (Thermo Fisher Scientific, Waltham, MA). After fixation, cells were washed three times with 1x PBS and blocked with 5% Bovine Serum Albumin (BSA; New England Biolabs, USA) with gentle shaking the coverslips in BSA at room temperature for 1 hour. After that the cells were incubated with primary antibody, anti-Integrin αvβ5 antibody, clone EM09902, ZooMAb® Rabbit Monoclonal (Sigma-Aldrich, St. Louis, MO), overnight at 4 °C followed by carefully washing of cells three times with 1x PBS. Then, the cells were incubated with fluorescent secondary antibody, Alexa Fluor 488 goat anti-rabbit IgG (Invitrogen, Thermo Fisher Scientific, Waltham, MA), for 1 hour at 4 °C in the dark condition. Again, the cells were washed three times with 1x PBS. Finally, the cells were stained with 4′,6-Diamidino-2-phenylindole (DAPI; Thermo Fisher Scientific, Waltham, MA) for 10 minutes. At last, the coverslips were taken out and Images (shown below) were acquired using a Cytation 5 Cell Imaging Multimode Reader (Agilent Technologies, Santa Clara, CA).

***Figure S10.*** *Representative immunofluorescence images showing cell-surface αvβ5 integrin expression in nonpermeabilized HeLa cells. (A) Bright-field image, (B) DAPI-stained nuclei (blue), (C) αvβ5 integrin immunofluorescence (green), and (D) merged DAPI and αvβ5 images. Boxed regions are enlarged in the upper-right insets. Scale bars = 200µm.*

# **S12. Influence of αvβ5 antibody blocking and cholesterol depletion on integrin-RGD mechanical interactions**

***Figure S11.*** *(A) Quantification of average number of beads bound per cell for RGD-integrin under different conditions. (B) Violin plots of rupture forces between integrin-RGD interactions on cells treated with anti-αvβ5* *antibody (right, F_avg_ = 27.9 pN), on healthy cells (middle, F_avg_ = 33.9 pN, taken from Figure 2B), and on cells with 10 µM methyl-β-cyclodextrin (MβCD) (right, n = 43, F_avg_ = 30.2 pN). (C) Bar diagram showing the average force for RGD-integrin at different conditions. “n” represents the number of cells.* *Error bars depict the standard deviation. p-value represents the statistical significance between different conditions.*

**S13. Effect of Perfluorooctanoic acid (PFOA) on RGD-integrin interaction**

Magnetic tweezers experiments were performed as described in Section S7 of the Supporting Information, both in the presence and absence of PFOA. For the PFOA-treated group, HeLa cells were first incubated in the experimental chamber for 5 hours and then treated with 150 μM PFOA (Sigma-Aldrich, St. Louis, MO) in complete DMEM for 1 hour before force measurements. Compared with untreated control, PFOA treatment significantly increased the average rupture force from 34.8 to 41.3 pN (*p* = 0.015).

***Figure S12.*** *Distribution of integrin-RGD rupture forces measured in control cells and cells treated with PFOA. Violin plots show individual rupture events (black dots) and average rupture forces (red stars). Treatment with 150 μM PFOA significantly increased the average rupture force compared with untreated controls, indicating enhanced integrin-mediated adhesion strength. Statistical significance was indicated by p value (student’s t- test).*

# **S14. Literature-derived K_d_ values used for qualitative comparison with magnetic tweezers rupture forces.**

**Table S6.** Literature-derived K_d_ values used for qualitative comparison with magnetic tweezers rupture forces.

| **Interaction** | **Reported K_d_ Value** | **Averaged K_d_ value** | **Assay Method** | **Experimental condition** |
| --- | --- | --- | --- | --- |
| Integrin-RGD | 3.87 nM for RAFT-RGD; 340 nM for cRGD | 171.912 nM | Fluorescence correlation spectroscopy (FCS) | Interaction assays were performed at RT in HBSS containing Mg^2+^ and Ca^2+^. One to 40 nmol/l of soluble integrin αvβ3 (CC1021, Chemicon) were mixed with 0.6 nmol/l of RAFT-RGD-Cy5 and RAFT-RAD-Cy5 or 2.4 nmol/l of cRGD-Cy5. (Ref. 38) |
| Nucleolin-AS1411 | 34.252 nM | 34.252 nM | Microscale thermophoresis (MST) | DPBS supplemented with 5 mM MgCl₂, 4.5 g/L glucose, 1 mg/mL BSA, and 0.1 mg/mL yeast tRNA. (Ref. 37) |
| Membrane-Cholesterol | 0.01-0.028 M | 0.019 M | mathematical fitting of published membrane-protein activity versus cholesterol-content isotherms | Apparent cholesterol affinity/partitioning in a membrane context, not conventional soluble ligand–receptor (Ref. 39) |

**References**

(1) Messner, J. J.; Glenn, H. L.; Meldrum., D. R. Laser-fabricated cell patterning stencil for single cell analysis. *BMC biotechnol.* **2017**, *17*, 89.

(2) Agard, N. J.; Prescher, J. A.; Bertozzi, C. R. A Strain-Promoted [3 + 2] Azide−Alkyne Cycloaddition for Covalent Modification of Biomolecules in Living Systems. *J. Am. Chem. Soc.* **2004**, *126*, 15046-15047.

(3) Liu, C.; Kubo, K.; Wang, E.; Han, K. S.; Yang, F.; Chen, G.; Escobedo, F. A.; Coates, G. W.; Chen, P. Single polymer growth dynamics. *Science* **2017**, *358*, 352-355.

(4) Zuo, L.; Ji, J.; Pokhrel, P.; Pokhrel, B.; Ren, K.; Mao, H.; Shen, H. Mechano-Electron Spin Coupling Modulates the Reactivity of Individual Coronazymes. **2023**.

(5) Marcus, J. M.; Burke, R. T.; DeSisto, J. A.; Landesman, Y.; Orth, J. D. Longitudinal tracking of single live cancer cells to understand cell cycle effects of the nuclear export inhibitor, selinexor. *Sci. Rep.* **2015**, *5*, 14391.

(6) Hovanessian, A. G.; Soundaramourty, C.; Khoury, D. E.; Nondier, I.; Svab, J.; Krust, B. Surface Expressed Nucleolin Is Constantly Induced in Tumor Cells to Mediate Calcium-Dependent Ligand Internalization. *PLoS ONE* **2010**, *5*, e15787.

(7) Bevington, P. R.; Robinson, D. K. *Data Reduction and Error Analysis for the Physical Sciences*; McGraw-Hill, **2003**.

(8) Ma, Y.; Leng, C.; Wang, H. Optimal Subsampling Bootstrap for Massive Data. *J. Bus. Econ. Stat.* **2024**, *42*, 174-186.

(9) Berditchevski, F. Complexes of tetraspanins with integrins: more than meets the eye. *J. Cell Sci.* **2001**, *114*, 4143-4151.

(10) Hu, X.; Roy, S. R.; Jin, C.; Li, G.; Zhang, Q.; Asano, N.; Asahina, S.; Kajiwara, T.; Takahara, A.; Feng, B.; et al. Control cell migration by engineering integrin ligand assembly. *Nat. Commun.* **2022**, *13*, 5002.
